# Exercise-downregulated CD300E acted as a negative prognostic implication and tumor-promoted role in pan-cancer

**DOI:** 10.3389/fimmu.2024.1437068

**Published:** 2024-07-31

**Authors:** Zhiwen Luo, Jinguo Zhu, Rui Xu, Renwen Wan, Yanwei He, Yisheng Chen, Qing Wang, Shuo Chen, Shiyi Chen

**Affiliations:** ^1^ Department of Sports Medicine, Huashan Hospital, Fudan University, Shanghai, China; ^2^ Department of Orthopaedics, Nantong Tongzhou Hospital of Traditional Chinese Medicine, Tongzhou, Jiangsu, China; ^3^ The First Clinical Medicine College, Nanjing Medical University, Nanjing, China; ^4^ Department of Orthopaedics, Kunshan Hospital of Chinese Medicine, Kunshan, Jiangsu, China; ^5^ Department of Sports Medicine, Nanjing Hospital of Chinese Medicine Affiliated to Nanjing University of Chinese Medicine, Nanjing, China

**Keywords:** physical exercise, breast cancer, CD300E, pan-cancer, bioinformatics, proliferation

## Abstract

**Background:**

Breast cancer ranks as one of the most prevalent malignancies among women globally, with increasing incidence rates. Physical activity, particularly exercise, has emerged as a potentially significant modifier of cancer prognosis, influencing tumor biology and patient outcomes.

**Methods:**

Using a murine breast cancer model, we established a control and an exercise group, where the latter was subjected to 21 days of voluntary running. RNA Sequencing, bioinformatics analysis, pan-cancer analysis, and cell experiments were performed to validate the underlying mechanisms.

**Results:**

We observed that exercise significantly reduced tumor size and weight, without notable changes in body weight, suggesting that physical activity can modulate tumor dynamics. mRNA sequencing post-exercise revealed substantial downregulation of CD300E in the exercise group, accompanied by alterations in critical pathways such as MicroRNAs in cancers and the Calcium signaling pathway. Expanding our analysis to a broader cancer spectrum, CD300E demonstrated significant expression variability across multiple cancer types, with pronounced upregulation in myeloma, ovarian, lung, and colorectal cancers. This upregulation was correlated with poorer prognostic outcomes, emphasizing CD300E’s potential role as a prognostic marker and therapeutic target. Moreover, CD300E expression was associated with cancer cell proliferation and apoptosis.

**Conclusion:**

The study highlights the dual role of exercise in modulating gene expression relevant to tumor growth and the potential of CD300E as a target in cancer therapeutics. Further research is encouraged to explore the mechanisms by which exercise and CD300E influence cancer progression and to develop targeted strategies that could enhance patient outcomes in clinical settings.

## 1 Introduction

Breast cancer, a predominant malignancy among women, has witnessed an increasing global incidence (1, 2). The World Health Organization reports that it stands as one of the leading causes of cancer-related deaths among women worldwide (3, 4). The impacts of breast cancer extend beyond severe health threats; its cells invade surrounding tissues and metastasize via lymphatic and circulatory systems to distant organs such as bones, liver, lungs, and brain, complicating and escalating the complexity of treatment protocols (5, 6). Additionally, the socioeconomic repercussions are profound, imposing substantial financial burdens during treatment and straining familial and social relationships due to the psychological toll of the disease (7, 8). Therefore, deepening our understanding of the mechanisms underlying breast cancer pathogenesis and developing innovative targeted therapies are imperative (9–11).

The beneficial impacts of physical activity on health and cancer prevention are multifaceted (12). Exercise enhances cardiovascular efficiency and muscle strength, augments bone density, and aids in osteoporosis prevention (13). It also boosts metabolism, which helps maintain a healthy weight and physique. Immunologically, physical activity increases lymphocyte counts, thereby strengthening the immune system’s defense against diseases, including cancer (14). Exercise also alleviates psychological stress and mitigates symptoms of anxiety and depression, enhancing overall mood and well-being, thereby indirectly reducing cancer risk (15–17). Persistently engaging in physical activities has been shown to correlate with lower cancer incidence rates, likely due to enhanced antioxidative capacity and expedited elimination of carcinogens (18–20). Recent research further underscores the therapeutic potentials of exercise in oncology. A study by Luo et al. revealed that physical activity could transform the immunological microenvironment of non-small cell lung cancer from a “cold” to a “hot” state, indicating that exercise not only increases the population of CD8+ T cells and M1 macrophages but also reduces immunosuppressive cells, thereby sensitizing tumors to immunotherapy (21). This transformative potential of exercise offers a promising adjunct to conventional cancer treatments, suggesting that integrating physical activity could significantly enhance therapeutic outcomes.

The CD300E gene encodes a protein that interacts with the TYRO protein tyrosine kinase binding protein, and is considered an activating receptor (22). Within the immune system, CD300E is posited to play a pivotal role in modulating the activity and functionality of immune cells (23–25). Research indicates that mCD300E can recognize sphingomyelin, thereby regulating the functions of atypical and intermediate monocytes through FcRγ and DAP12 (26). In the realm of oncology, the study of CD300E is garnering increasing attention due to its potential role in modulating tumor immune responses and facilitating immune escape (25, 27). Specifically, CD300E may promote tumor growth and dissemination by influencing the interactions between tumor cells and the immune system. In addition, one patent have reported that CD300E siRNA delays or halts cancer progression by blocking or knocking down cd300e to inhibit its activity or expression, and that the rate of tumor growth is significantly inhibited in mouse tumors compared to controls. Understanding the precise mechanisms of CD300E’s involvement in tumor immunity is critical for the development of novel immunotherapeutic strategies, which could include modulating its expression or function to enhance the immune system’s capacity to target tumors (28, 29).

This study has identified CD300E as a critical target through gene sequencing of voluntary running wheel exercises in mice as an anti-breast cancer initiative. By further analyzing CD300E through bioinformatics and cellular biology experiments, we aim to explore and demonstrate its role in tumor development and progression. This research not only sheds light on the mechanistic underpinnings of CD300E in cancer biology but also underscores the potential of exercise-induced molecular responses as a strategic approach in cancer prevention and treatment.

## 2 Materials and methods

### 2.1 Cell culture

The 4T1 mouse cancer cell line (catalog KGG2224-1) and MDAMB231 (catalog KGG3220-1) were procured from KeyGEN (Nanjing, China). MDA-MB-468 was procured from FengHui ShengWu, China. 4T1 cells were cultured in RPMI-1640 medium enriched with 10% fetal bovine serum (FBS) and sustained at 37°C in either an ambient atmosphere or one containing 5% CO_2_. MDAMB231 and MDAMB468 cells were cultured in the MEM media with 1% non-essential amino acid and 1 mM sodium pyruvate. All media were added with 10% FBS at 37°C with or without 5% CO_2_.

### 2.2 Animal interventions

Female BALB/c mice, aged 5-6 weeks, were obtained from the Shanghai Laboratory Animal Center (SLAC). To establish a triple-negative breast cancer (TNBC) model, 4T1 cells (5 × 10^6) were subcutaneously injected into the abdomen of BALB/c mice. The choice of this specific strain and demographic was based on its relevance to breast cancer research and its consistent response to exercise interventions. All mice were in good health, verified by a veterinarian prior to the commencement of the study. The mice were housed in a controlled environment with a 12-hour light/dark cycle, and were given free access to food and water. Tumor growth was monitored and measured regularly every 2-3 days using calipers. Mice were randomly divided into two groups: an exercise group (E) and a non-exercise group (NE), each comprising five animals. The exercise group underwent a 21-day regimen of voluntary running (no speed or distance limitation), whereas the non-exercise group was maintained under normal husbandry conditions without dietary restrictions. After 21 days, the mice were euthanized, and tumor tissues were collected for mRNA sequencing analysis. Animal experiments were granted by Ethics Committees at Nanjing Medical University.

### 2.3 mRNA sequencing and bioinformatics analysis

21 days subsequent to administering the treatments, tumor samples from mice were carefully collected for mRNA sequencing analysis (30). Following various treatments, cell samples were diligently harvested. The extraction of total RNA from these samples was performed using the highly regarded RNeasy Mini Kit (Qiagen, Hilden, Germany). After RNA extraction, the construction of paired-end libraries was carried out using the TruSeq RNA Sample Preparation Kit (Illumina, USA), adhering meticulously to the protocol provided by TruSeq RNA Sample Preparation. The Shanghai Biotechnology Corporation was tasked with the responsibility of constructing and sequencing the libraries. For the precise mapping of clean reads to the Rnor 6.0 reference genome, allowing up to two mismatches, the widely acclaimed Hisat2 software (version 2.0) was utilized. Subsequent to genome mapping, the esteemed Stringtie software (version 1.3.0) was employed to generate and annotate Fragments per kilobase of exon per million (FPKM) values. Gene expression data were normalized using the trimmed mean of M-values (TMM) method to correct for library size differences and compositional biases. Top-10 genes were shown.

Statistical significance was determined with a P-value threshold set according to the false discovery rate (FDR). mRNAs exhibiting a fold change of ≥ 2 and an FDR ≤ 0.05 were identified as differentially expressed. To further investigate the biological pathways involved, meticulous KEGG pathway analysis was performed using the revered KEGG database (http://www.genome.ad.jp/kegg) within the R environment. Additionally, Gene Set Enrichment Analysis (GSEA) was conducted using R BiocManager to delve deeper into the molecular mechanisms influenced by the treatments.

### 2.4 Pan-cancer analysis

#### 2.4.1 Gene expression and datasets obtained

We utilized the Human Protein Atlas (HPA) to collate comprehensive RNA and protein expression profiles of CD300E in human samples. Furthermore, detailed data on CD300E expression across various tissues and cell lines were sourced from the Harmonizome database. We expanded our dataset by incorporating CD300E mRNA expression data from cancerous, paracancerous, and normal tissue samples provided by TCGA and GTEx databases. Our study spanned a diverse set of 33 cancer types including, Adrenocortical carcinoma (ACC), Bladder Urothelial Carcinoma (BLCA), Breast invasive carcinoma (BRCA), Cervical squamous cell carcinoma and endocervical adenocarcinoma (CESC), Cholangiocarcinoma (CHOL), Colon adenocarcinoma (COAD), Lymphoid Neoplasm Diffuse Large B-cell Lymphoma (DLBC), Esophageal carcinoma (ESCA), Glioblastoma multiforme (GBM), Head and Neck squamous cell carcinoma (HNSC), Kidney Chromophobe (KICH), Kidney renal clear cell carcinoma (KIRC), Kidney renal papillary cell carcinoma (KIRP), Acute Myeloid Leukemia (LAML), Brain Lower Grade Glioma (LGG), Liver hepatocellular carcinoma (LIHC), Lung adenocarcinoma (LUAD), Lung squamous cell carcinoma (LUSC), Mesothelioma (MESO), Ovarian serous cystadenocarcinoma (OV), Pancreatic adenocarcinoma (PAAD), Pheochromocytoma and Paraganglioma (PCPG), Prostate adenocarcinoma (PRAD), Rectum adenocarcinoma (READ), Sarcoma (SARC), Skin Cutaneous Melanoma (SKCM), Stomach adenocarcinoma (STAD), Testicular Germ Cell Tumors (TGCT), Thyroid carcinoma (THCA), Thymoma (THYM), Uterine Corpus Endometrial Carcinoma (UCEC), Uterine Carcinosarcoma (UCS), Uveal Melanoma (UVM).

For statistical analysis, we utilized R software (version 4.2.2) and employed the ggplot2 package to depict CD300E expression across the cancer spectrum. We adopted the median expression level as the threshold for differential expression analyses. Differences between expression groups were assessed using the Wilcoxon rank-sum test.

#### 2.4.2 Survival analysis of CD300E in the 33 cancers

We also conducted survival analyses to explore the prognostic potential of CD300E expression in cancer (18). Using the survival package in R, we performed Kaplan-Meier analyses and employed Cox regression to compare survival outcomes between groups with high and low expression of CD300E. The impact of CD300E expression on survival was visually represented through forest plots using the survminer and ggplot2 packages.

#### 2.4.3 Genetic alteration analysis of CD300E

An investigation into the genetic alterations associated with CD300E was conducted through the cBioPortal. This analysis included an examination of somatic mutation frequencies and detailed genomic information, helping to elucidate the mutation landscape of CD300E in various cancers.

#### 2.4.4 Immunogenomic analyses of CD300E in the 33 cancers

In our immunogenomic analysis across 33 different cancers, we utilized the “GSVA” package and the “ssGSEA” algorithm to assess the relationship between CD300E expression and various immune components, including tumor-infiltrating lymphocytes, immunostimulators, immunoinhibitors, MHC molecules, chemokines, and chemokine receptors. The correlations were determined using Spearman’s correlation coefficient, and p-values less than 0.05 were deemed significant. To effectively display these correlations, we generated heatmaps using the “ggplot2” package.

#### 2.4.5 Functional enrichment analysis of CD300E

We carried out Gene Ontology (GO) and Kyoto Encyclopedia of Genes and Genomes (KEGG) pathway enrichment analyses to examine the functions and pathways associated with genes interacting closely with CD300E. These genes were identified through STRING and analyzed using the “clusterProfiler” and “org.Hs.eg.db” packages in R. We set a stringent cutoff threshold of a p-value < 0.01 for both GO and KEGG enrichment analyses. The outcomes of these analyses were visually represented using bubble charts created with the “ggplot2” package.

### 2.5 Cellular experiments

#### 2.5.1 Knockdown of CD300E gene

To knock down CD300E gene expression in tumor cells, we designed and synthesized small interfering RNAs (siRNAs) targeting CD300E using *In vivo*gen-based method (Detailed sequencing can be found in the **Supplementary Table 1**). These siRNA sequences were algorithmically predicted and selected as the most likely to effectively target CD300E mRNA. The specific steps are as follows: siRNA transfection: siRNA transfection was performed using Lipofectamine 2000 (Invitrogen) according to the manufacturer’s instructions. Briefly, cells were incubated with a mixture of Lipofectamine 2000 and siRNA to form a complex 24 hours after inoculation and then added to the cells. Gene knockdown efficiency assessment: 48 hours after transfection, CD300E mRNA and protein levels were analyzed by real-time quantitative PCR (qPCR) to verify the efficiency of siRNA knockdown.

#### 2.5.2 Overexpression of CD300E gene

cDNAs of mouse CD300e (GenBankTM accession number NM_172050.3) were isolated by PCR from a cDNA library of mouse BM cells. To overexpress CD300E, we constructed a plasmid containing the complete CD300E coding region. This plasmid drives the expression of CD300E under the control of CMV promoter. The steps of the overexpression experiment are as follows:

Plasmid construction: the cDNA of CD300E was cloned into the expression vector pCMV, and the correctness of the insert sequence was verified by gene sequencing. Plasmid transfection: transfection of plasmid DNA was performed using Lipofectamine 2000. Cells were transfected 24 hours after inoculation, following similar steps as described above for siRNA transfection. Expression verification: 48 hours after transfection, mRNA and protein expression of CD300E were detected by qPCR to confirm the effect of gene overexpression.

#### 2.5.3 Proliferation/apoptosis/migration/invision

To evaluate the proliferation of cancer cells, we cultured the cells in suspension and then seeded them at a density of 5 × 10^3 cells/mL (100 μL per well) in a 96-well plate. The plate was maintained at 37°C. Subsequently, we added 10 μL of CCK-8 reagent (catalog KGA9305, KeyGEN, Nanjing, China) to each well and allowed the plate to incubate for two hours before measuring the optical density at 450 nm using a microplate reader.

For the assessment of cell migration and invasion, we utilized Transwell chambers, applying a Matrigel coating for invasion assays and no coating for migration assays. We introduced cancer cells (5×10^4) in 200 μL of serum-free medium into the upper chamber, while the lower chamber was filled with 600 μL of medium supplemented with 10% FBS.

To determine levels of cell apoptosis, we analyzed the apoptosis rate using an Annexin V-FITC/PI Kit (Cat. KGA1102, KeyGEN, Nanjing, China), following the protocol provided by the manufacturer. This method facilitated a precise evaluation of the apoptotic stages within the cancer cell populations.

#### 2.5.4 Real-time quantitative polymerase chain reaction

To assess mRNA abundance at the cellular level, total RNA was meticulously extracted from cells and muscle tissues using the Trizol reagent (Invitrogen) and was precisely quantified with a Nanodrop instrument (Thermo Scientific, USA). Following this, cDNA was synthesized and served as a template for quantifying mRNA expression levels in quantitative PCR (qPCR) assays. These assays were performed using the TB Green™ Premix Ex Taq™ II kit (Takara; RR820A), with GAPDH used as an internal control for normalization. Specific qPCR primers, essential for the amplification of mRNA, were synthesized by Bioengineering (Shanghai, China). The relative expression levels of the mRNA in each sample were calculated using the comparative Ct method (2^-ΔΔCt), ensuring the accuracy of the results through at least three independent experimental replicates. To provide a consistent baseline for comparison, all values were normalized against the control condition. Details of the primer sequences used are available in **Supplementary Table 1**.

### 2.6 Statistical methods

Statistical analysis and figure generation were performed with R language version 4.0.2 and Graphpad Prism 9.0. For the comparison of continuous variables between two groups, the choice between the Student t-test and the Mann-Whitney test depended on specific conditions. When comparing multiple groups, either one-way ANOVA or the Kruskal-Wallis test with subsequent multiple comparisons was used, depending on the circumstances. The prognostic significance of categorical variables was determined using the log-rank test. Statistical significance was set at a P value <0.05 across all analyses.

## 3 Results

### 3.1 Impact of voluntary running on tumor growth and gene expression

**Figure 1:**
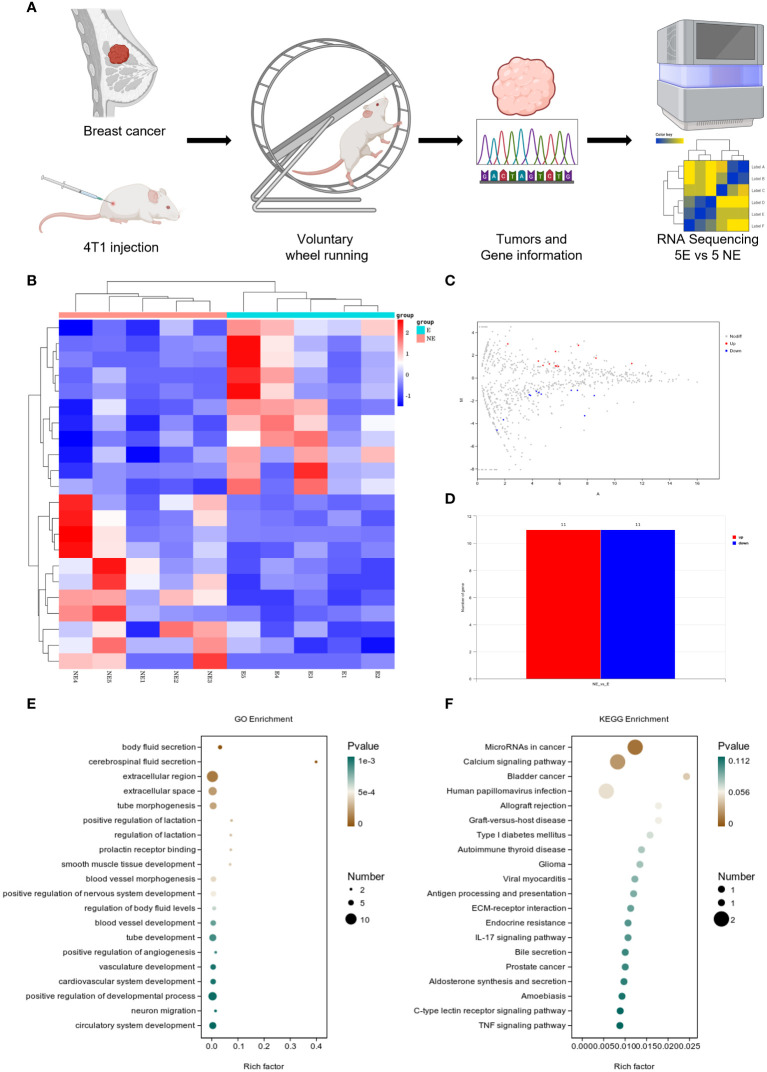
Voluntary wheel running exercise inhibits breast cancer growth. **(A)** Schematic diagram of the experiment. **(B)** Heatmap of Hierarchical clustering analysis of changed mRNAs. **(C, D)** Volcano plot and column of mRNAs differentially expressed between NE and E group. n = 5. **(E, F)** Bubble plot showing GO and KEGG enrichment by all the differentially expressed mRNAs expressed in tumors, including biological process, cellular component, and molecular function.

Following the intervention of exercise, a significant reduction in tumor size and weight was observed at day 21, with minimal changes in the body weight of the mice (**Supplementary Figures 1A, B**). We then conducted mRNA sequencing analysis on five matched pairs (**Figure 1A**). The quality control results confirmed normal parameters, with high intra-group consistency and notable expression differences between groups (**Supplementary Figures 2A–E**). Volcano plots and heatmaps revealed differential expression of 22 genes, among which CD300E expression was significantly reduced in the exercise group (E), representing only 46% of that in the non-exercise group (NE), with a p-value of 0.008 (**Figures 1B–D**). Gene enrichment analysis highlighted significant alterations in extracellular components, with the most pronounced changes observed in the MicroRNAs in cancers and Calcium signaling pathway (**Figures 1E, F**).

### 3.2 Pan-cancer analysis

#### 3.2.1 Expression variability of CD300E in pan-cancer

**Figure 2:**
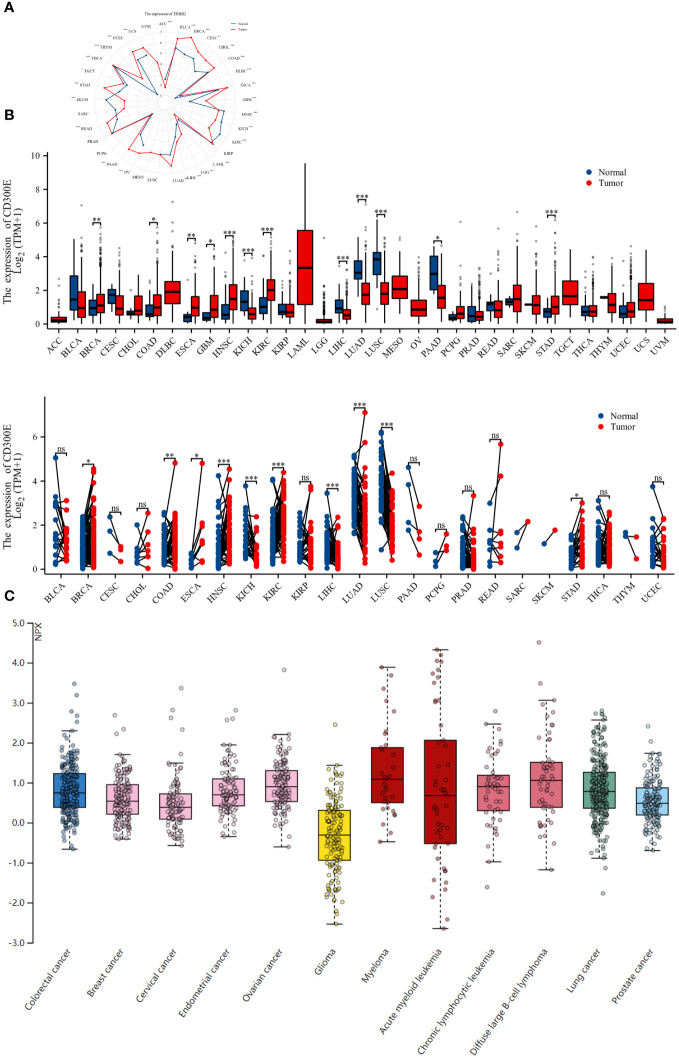
Differential expression pattern of CD300E. **(A)** Differential CD300E mRNA expression between paired samples in TCGA cancers. The red dot represents cancer samples, and the blue dot represents paired normal samples. Radargrams visualize and compare CD300E expression in different tumors. *p < 0.05, **p < 0.01, and ***p < 0.001. **(B)** Differential CD300E mRNA expression between TCGA cancers and GTEX normal tissues. The red column represents cancer samples, and the blue column represents normal samples. The normal group was normal tissue in TCGA and GTEX databases. *p < 0.05, **p < 0.01, and ***p < 0.001. **(C)** CD300E protein expression in different cancer types in Human Atlas.

To evaluate the expression of CD300E mRNA in normal human tissues, we analyzed data from the GTEx, HAP, and Consensus datasets. CD300E showed higher expression in tissues such as blood, lung, bone marrow, appendix, and bladder (**Supplementary Figure 1**). Further in-depth evaluation using RNA-seq data from TCGA and GTEx databases revealed significant expression differences in CD300E across 33 types of cancer. In unmatched samples (**Figure 2A**), CD300E was notably upregulated in cancers like BRCA, COAD, ESCA, GBM, HNSC, KIRC, and STAD, and downregulated in KICH, LIHC, LUAD, LUSC, and PAAD. In matched samples (**Figure 2B**), upregulation was significant in BRCA, COAD, ESCA, HNSC, KIRC, and STAD, while downregulation was noted in COAD, KICH, LIHC, LUAD, and LUSC. The Human Atlas database further assessed the protein expression of CD300E across various cancers, showing upregulation in Myeloma, Diffuse large B-cell lymphoma, Ovarian cancer, Lung cancer, and Colorectal cancer without significant downregulation in any cancer type (**Figure 2C**).

#### 3.2.2 Prognostic impact of CD300E in pan-cancer

**Figure 3:**
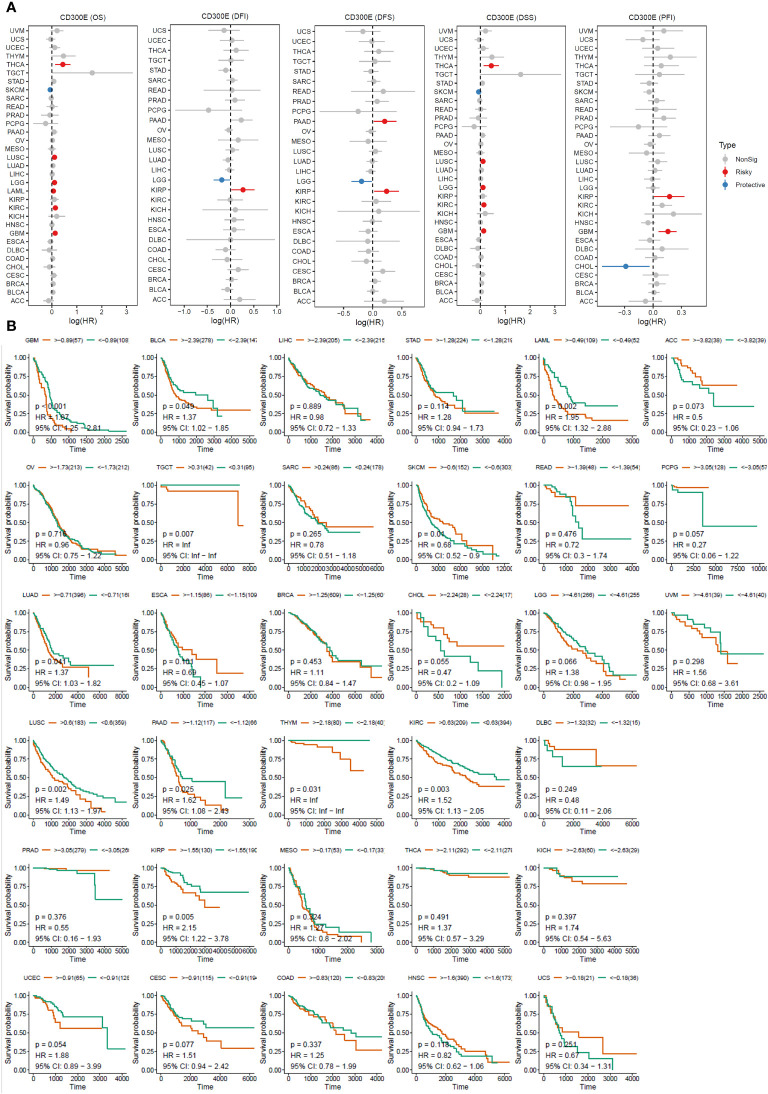
High expression of CD300E reduced patient survival period. **(A)** Forest plot of hazard ratios (HR) for overall survival (OS), PFI, DSS, DFS, and DFI for different cancer types associated with CD300E expression. Dots indicate log-transformed hazard ratios, red indicates significant risk, blue indicates protective associations, and gray indicates non-significant associations. **(B)** Individual OS figures for each cancer type.

For overall survival (OS) and disease-specific survival (DSS), CD300E posed a risk factor in THCA, LUSC, LGG, LAML, KIRC, and GBM, while it acted as a protective factor only in SKCM (**Figures 3A, B**). For disease-free interval (DFI), progression-free interval (PFI), and disease-free survival (DFS), CD300E was a risk factor in KIRP, PAAD, and GBM, and a protective factor in LGG and CHOL (**Figure 3A**).

#### 3.2.3 Correlation analysis of CD300E in pan-cancer

**Figure 4:**
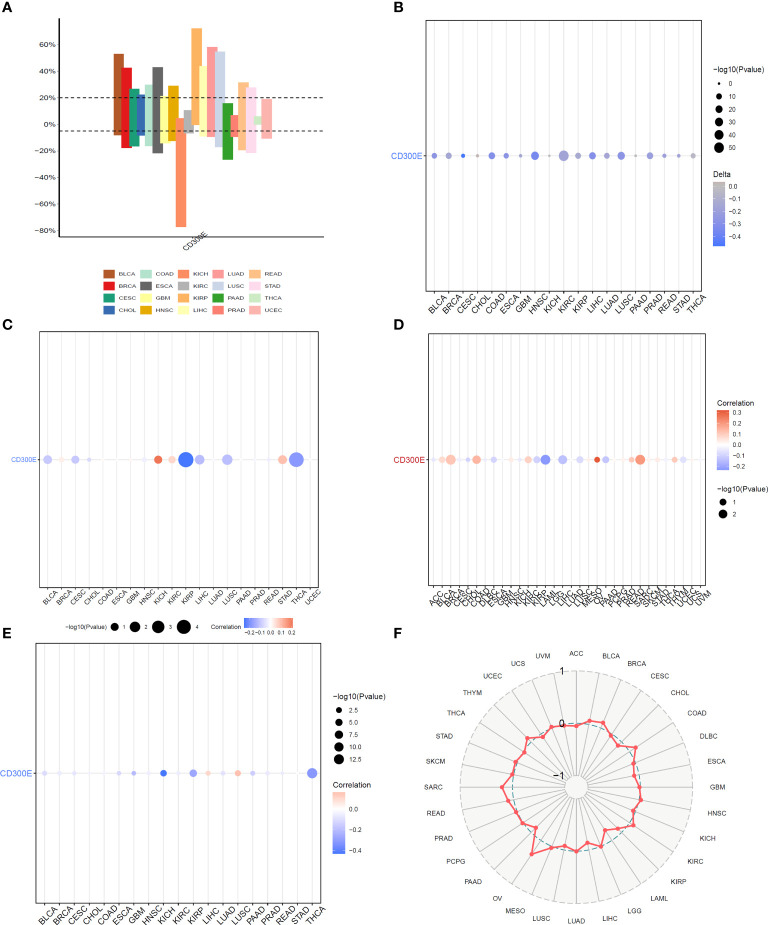
Correlation analysis of CD300E in pan-cancer. **(A)** Bar graphs illustrate CD300E copy number variation in different cancers. **(B)** CD300E copy number and pan-cancer direct correlation analysis. **(C, D)** The correlation between the methylation status of gene promoter regions and CD300E in multiple cancer types **(E, F)** The correlation between tumor mutational burden (TMB) and CD300E expression.

Copy number variations (CNVs), a common form of genomic instability in cancer, can lead to altered gene expression affecting cell proliferation, differentiation, and death. Bar graphs (**Figure 4A**) showed changes in CD300E copy numbers across various cancers, with significant variations in KICH and READ. Further correlation analysis indicated a negative relationship between CD300E copy numbers and cancer progression in KIRP and THCA, and a positive correlation in KICH and STAD (**Figure 4B**). Promoter methylation, a critical epigenetic regulatory mechanism affecting gene expression without altering the DNA sequence, was analyzed to explore its relationship with CD300E expression across multiple cancer types. Both unmatched and matched tumor samples showed a negative correlation between CD300E expression and methylation, particularly in KIRC and THCA (**Figures 4C, D**). Additionally, the relationship between tumor mutational burden (TMB) and CD300E expression was investigated, revealing a positive correlation in SARC, OV, COAD, BRCA, BLCA, and THYM, and a negative correlation in LAML, LIHC, and PAAD (**Figures 4E, F**).

#### 3.2.4 Analysis of CD300E on the immune microenvironment across cancers

**Figure 5:**
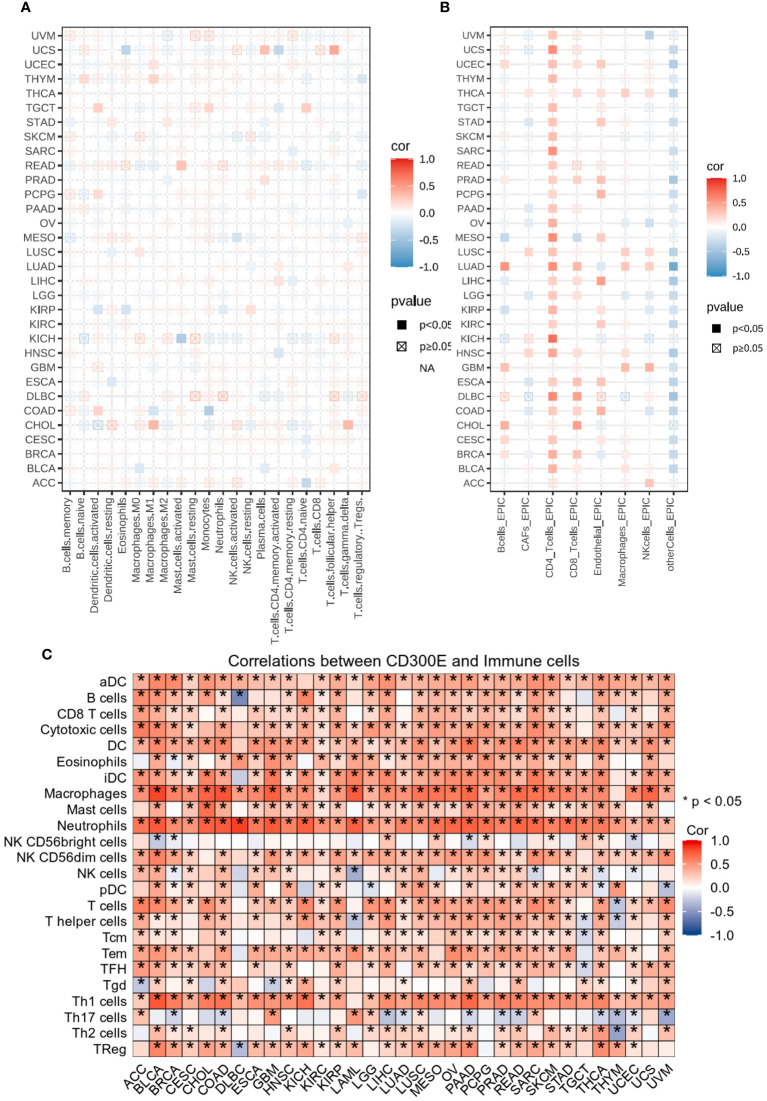
Analysis of immune microenvironmental cellular regulation of pan-cancer by CD300E. **(A)** Heatmap of immune cell infiltration in pan-cancer analyzed using the Cibersort method. Each cell represents the correlation between CD300E expression and the level of a specific immune cell type, and the intensity and sign of the color correspond to the strength and direction of the correlation, respectively. Statistical significance is indicated by the box around the cell. **(B)** CD300E pan-cancer immuno-infiltration analysis using Cibersort. **(C)** Gene Commons data analysis of correlations between single genes and immune infiltration results, using heatmap format to present results.

Heatmap analysis from **Figure 5A** intricately details the correlations between CD300E expression and various immune cell subtypes across different types of cancers. Notably, in cancers such as BRCA (Breast Cancer) and COAD (Colorectal Adenocarcinoma), a significant positive correlation exists between CD300E expression and M2 macrophages, typically associated with a tumor-promoting immunosuppressive environment. This suggests that elevated expression of CD300E may foster an immunosuppressive state conducive to tumor growth and metastasis. Conversely, in Lung Adenocarcinoma (LUAD), CD300E exhibits a negative correlation with natural killer (NK) cells, although this association generally lacks statistical significance. This trend implies that in certain cancer contexts, CD300E expression may inversely affect the immunosurveillance capabilities of NK cells, potentially contributing to mechanisms of immune escape. Additionally, in certain cancer types like BRCA, CD300E shows a positive correlation with regulatory T cells (Tregs), which play a critical role in modulating the immune system, particularly in maintaining immune tolerance and suppressing excessive immune responses. Increased CD300E expression might enhance the functionality of Tregs, thereby fostering an immune-suppressive tumor microenvironment favorable for tumor survival and progression.

EPIC analysis, a vital tool in studying the tumor microenvironment, enables researchers to understand the dynamic variations of different cell types within tumors, which is crucial for advancing tumor immunology and developing new therapeutic strategies (**Figure 5B**). From the heatmap, it is evident that CD300E’s correlations with various immune cells vary, illustrating the heterogeneity of tumor microenvironments. For instance, in breast and colorectal cancers, Cancer-associated fibroblasts (CAFs) show a strong positive correlation with CD300E expression, suggesting their significant role in supporting or enhancing tumor growth and invasion, closely linked with the expression of this gene. Moreover, in cancers like LUAD, the activity of CD8+ T cells significantly correlates with CD300E expression, reflecting their importance in the tumor immune response and the potential regulatory role of this gene. Further analysis using the TCGA database’s pan-cancer dataset revealed a broadly positive correlation between CD300E and various immune cells across different cancer types (**Figure 5C**).

#### 3.2.5 Pathway enrichment and key gene mutation analysis of CD300E across cancers

**Figure 6:**
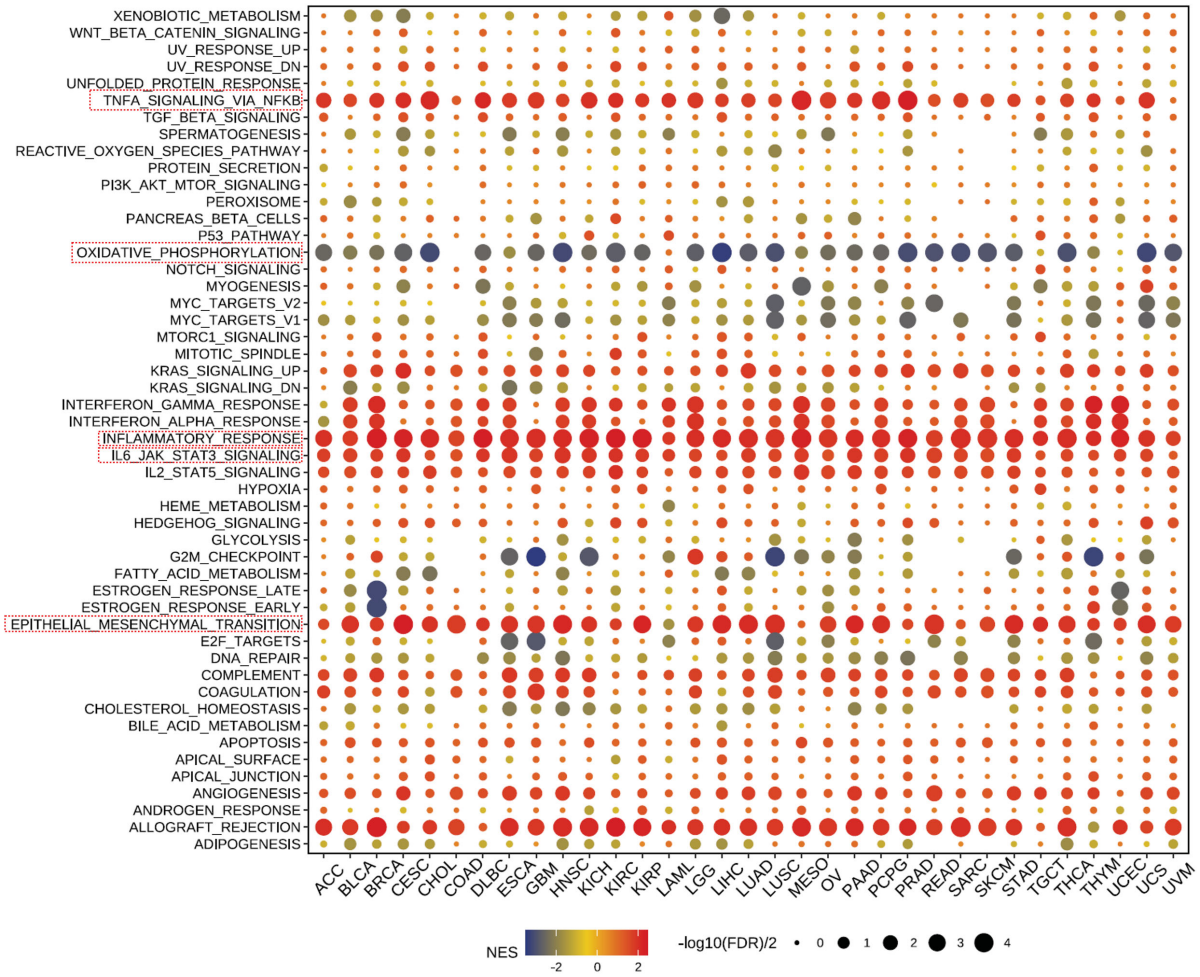
Pathway enrichment of CD300E in pan-cancer. Dot plots represent pan-cancer GSEA results using the official immunization gene set (GMT file) as a reference. Functional pathways are from GM7 files and are shown on the y-axis, with different cancer types shown on the x-axis. Dot color indicates correlation with CD300E expression; red indicates positive correlation and blue negative correlation. The size of the dots represents the -log10(FDR) value, indicating the significance of the enrichment.

Our further evaluation of CD300E’s function in pan-cancer contexts revealed significant findings via the GSEA methodology. CD300E notably suppresses oxidative stress pathways, potentially facilitating conditions favorable for tumor growth. Additionally, CD300E significantly enhances pathways such as TNF-α signaling, inflammatory response pathways, IL6-JAK signaling, and epithelial-mesenchymal transition (EMT), all of which are documented to potentially promote tumor growth and metastasis (**Figure 6**).

A heatmap depicting the frequency of key gene mutations across various cancers highlights the high mutation rates of genes such as TP53 in LUAD, APC in COAD, and PTEN in UCEC, indicating their common involvement in these cancers. Specific cancer types like BRCA, LGG, and HNSC show frequent mutations in genes like TP53, PIK3CA, and CDKN2A, providing insights that may guide therapeutic strategies (**Supplementary Figure 5A**).

### 3.3 Impact of CD300E on breast cancer cells

**Figure 7:**
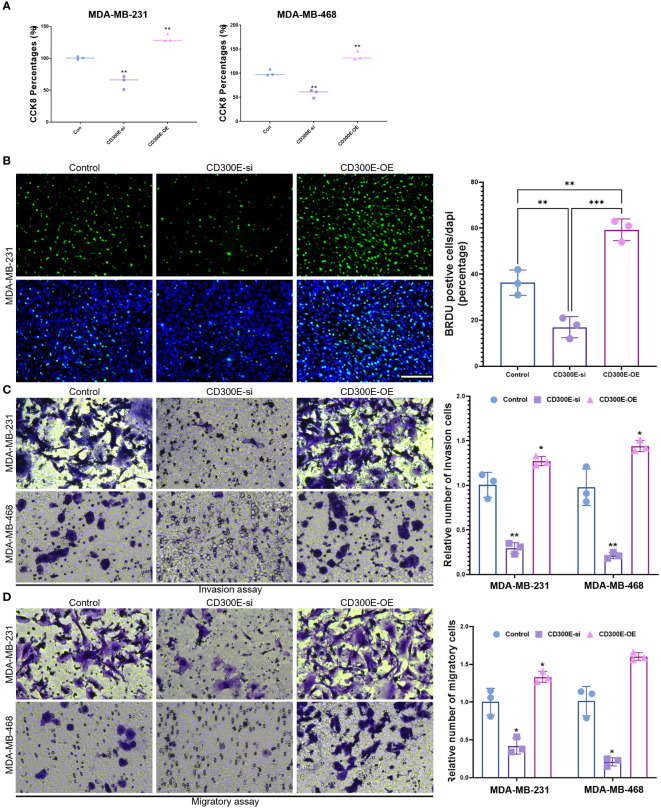
CD300E promotes breast cancer tumor cell growth. **(A)** The viability of control, CD300E-inhibited, and CD300E-overexpressed tumor cells was examined at 48h after transfection by CCK-8 assay. The statistical significance of the differences between various treatments is determined by one-way ANOVA with Bonferroni post-test (n = 3). Data are presented as mean ± SD. *P < 0.05 **P < 0.01. **(B)** The proliferative capacity of control, CD300E-inhibited, and CD300E-overexpressed tumor cells was examined at 24h after transfection by BRDU. The statistical significance of the differences between various treatments is determined by one-way ANOVA with Bonferroni post-test (n = 3). Data are presented as mean ± SD. *P < 0.05 **P < 0.01 ***P < 0.001. **(C, D)**. The migratory and invasive capacity of control, CD300E-inhibited, and CD300E-overexpressed tumor cells were examined at 24h after transfection by Boyden chamber assay. Total original magnification, 200×. The statistical significance of the differences between various treatments is determined by one-way ANOVA with Bonferroni post-test (n = 3). Data are presented as mean ± SD. *P < 0.05 **P < 0.01.

**Figure 8:**
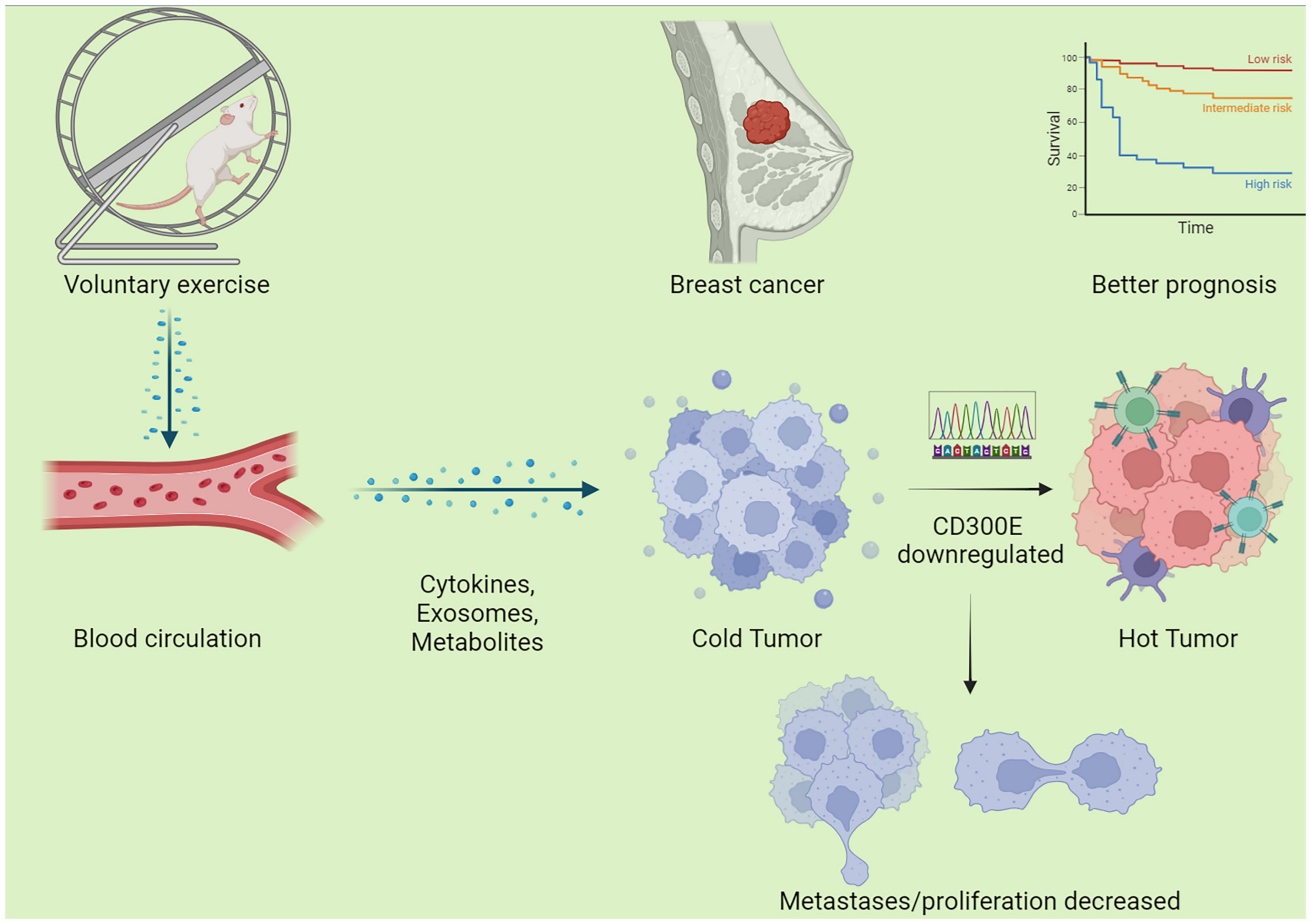
Schematic graph of this study. Exercise decreased CD300E expression of cells in breast cancer through a circulatory effect, which promotes immune cell infiltration, decreased tumor cell metastases/proliferation, warms the tumor microenvironment, and improves the prognosis of tumor patients.

Finally, our study delves into the cellular functions of CD300E. We validated the expression of the CD300E gene after siRNA or plasmids intervention (**Supplementary Figures 6A, B**). Compared to control cells, overexpression of CD300E in MDAMB468 and MDAMB231 breast cancer cells leads to increased proliferation and cell viability, while suppression of CD300E expression reduces proliferation and cell viability (**Figures 7A, B**). Furthermore, overexpression of CD300E significantly promotes the migratory and invasive capabilities of these tumor cells, whereas its inhibition reduces these properties (**Figures 7C, D**). Overall, targeting CD300E could directly inhibit tumor cells, significantly impeding cancer progression and presenting a novel therapeutic target (**Figure 8**).

## 4 Discussion

This research explored the impact of exercise on tumor growth and gene expression within a murine model, focusing particularly on the expression patterns, functions, and potential clinical significance of the CD300E gene across various cancers. Our findings indicate that CD300E may adversely affect prognosis and promote tumor progression across a range of cancers. Additionally, exercise appears to inhibit breast cancer progression potentially by downregulating CD300E.

Exercise as well as widespread is believed to promote human health and improve a wide range of diseases (31–33). The phenomenon of exercise against cancer has been widely explored in recent years, but there is still a large number of exercise-responsive molecules whose roles need to be explored (34–38). Our study confirmed the positive impact of physical activity on inhibiting tumor growth. Exercise intervention significantly reduced tumor size and weight in the murine model without markedly affecting body weight. These outcomes suggest that moderate physical activity might suppress tumor growth by modifying the tumor microenvironment or regulating specific signaling pathways. Analysis of differentially expressed genes revealed significant downregulation of CD300E in the exercise group, indicating its role in tumor growth regulation, particularly within an active context. Furthermore, gene enrichment analysis showed significant changes in extracellular components and associated signaling pathways, such as MicroRNAs in cancers and the Calcium signaling pathway, providing clues on how exercise might influence tumor biology through molecular mechanisms.

In our pan-cancer analysis, CD300E exhibits significant expression variability across multiple cancer types, underscoring its potential role in various malignancies. Notably, CD300E is upregulated in cancers such as Myeloma, Diffuse Large B-cell Lymphoma, Ovarian Cancer, Lung Cancer, and Colorectal Cancer, suggesting its involvement in the progression of these diseases. Prognostic analyses reveal that CD300E acts as a risk factor in several cancers, providing valuable insights that may guide clinical prognostic assessments and therapeutic decision-making. Studies on the variability of CD300E copy numbers and their correlation with tumor mutational burden offer critical perspectives on its role in cancer progression. These findings support the notion that CD300E may promote cancer development by impacting genetic stability and the interactions within the immune microenvironment.

Analysis of the relationships between CD300E and various immune cell subpopulations indicates that CD300E may influence tumor growth and immune escape by modulating immune cells within the tumor microenvironment, particularly immunosuppressive M2 macrophages and regulatory T cells. Past studies have also shown that CD300E and T cells are associated with the regulation of immune function in macrophages, and more mechanistic studies are needed to explore this (22–24). But, the results also illustrated that CD300E showed a significant positive correlation with most other immune-promoting immune cells, including CD8 T cells, neutrophils, NK cells, etc. The literature reports that these cells more or less affect the heating and cooling of the immune microenvironment. It has been reported in the literature that these cells more or less affect the heat and cold of the immune microenvironment (26). The increased expression of CD300E resulted in both Immunosuppressive and immunopromoting cells, affecting the tumor microenvironment, which could potentially affect the prognosis and the degree of response to immunotherapy. These insights lay a theoretical foundation for targeting CD300E in immunotherapeutic strategies.

Furthermore, our analysis elucidates the role of CD300E in regulating key signaling pathways related to cancer progression, especially in suppressing oxidative stress pathways and activating several pathways that promote tumor progression. The inhibition of oxidative stress pathways may provide cancer cells with mechanisms to evade programmed cell death, thereby covertly supporting tumor growth and survival (39–41). Concurrently, CD300E significantly activates pathways such as the TNF-α pathway, inflammatory response pathways, the IL6-JAK pathway, and the epithelial-mesenchymal transition pathway, all closely associated with the invasiveness and metastatic potential of tumors (42–45). These pathways’ activation might facilitate the dissemination of tumor cells within the host. Analysis of the frequency of key gene mutations reveals frequent mutations in genes such as TP53, APC, and PTEN across various cancers, highlighting these genes as critical factors in tumor development and progression (46). These mutations may affect cell cycle regulation, DNA repair mechanisms, and pathways of cell death, further substantiating the potential role of CD300E in pan-cancer contexts.

Additionally, our cellular experiments clearly demonstrate that the overexpression of CD300E in breast cancer cells is closely associated with enhanced cellular proliferation, reduced apoptosis rates, and increased migration and invasion capabilities. These findings not only confirm the role of CD300E as a tumor-promoting factor but also highlight its potential as a therapeutic target. Experiments aimed at inhibiting CD300E expression further validate its significant role in tumor cell proliferation and survival, offering a potential therapeutic strategy to curb the progression of breast cancer. Studies have reported that CD300E can modulate apoptosis in monocytes by affecting calcium channels, which is consistent with our biological predictions. In addition, altered calcium signaling affects the behavior of immune cells (including T cells and macrophages), influencing their activation and cytokine production, thereby altering the immune microenvironment (47–49). Therefore, we hypothesize that the ability of CD300E to promote tumor cell value-addition and migration is reached by regulating calcium channels.

Mechanically, how exercise regulates CD300E lowering this process was not explored in this study. However, a large body of literature has reported that exercise can bring about a series of physiological changes, including changes in the metabolome, proteins, and related molecules in the genome (12, 50, 51). Specifically, we hypothesize that exercise-induced changes in systemic factors, such as serum circulating exosome, muscle derived cytokines, and hormones, could impact transcription factors like NF-κB and STAT3, known regulators of gene transcription (41, 52–54). Additionally, the role of epigenetic modifications, including DNA methylation and histone acetylation, in the regulation of gene expression in response to physical activity could also influence the expression of CD300E (55). In addition, the direct upstream transcription factor(s) by which exercise regulates CD300E expression in tumor cells remains unknown. We proposed that exercise activates AMP-activated protein kinase (AMPK) and peroxisome proliferator-activated receptor gamma coactivator 1-alpha (PGC-1alpha), which are central to tumor cell expression (56, 57). These molecules may influence the transcription factors and co-regulators that control CD300E expression. Furthermore, exercise can also modulate the expression of cellular miRNA, which may post-transcriptionally regulate CD300E (58, 59). For example, miR-4270 has been reported to directly target CD300E (60), but these are speculations based on the literature, and future studies will need to further explore the mechanisms by which exercise regulates CD300E.

As for the clinical translational perspective, we believe that patients with high CD300E expression may benefit from more intensive or specific types of exercise therapies that are particularly effective in downregulating CD300E. Conversely, patients with low CD300E expression may require different exercise regimens or adjunctive therapies to achieve optimal results. To test these hypotheses, we recommend that future studies design clinical trials that stratify patients according to CD300E expression levels. These trials should include a variety of exercise regimens from moderate to high intensity and monitor changes in CD300E expression, tumor progression, and clinical prognosis. In addition, patient-reported outcomes and quality-of-life measures should be included to assess the broader impact of tailored exercise interventions. Moreover, we recommend longitudinal studies to track CD300E expression and tumor progression in response to sustained exercise therapy. These studies will help determine the sustainability of exercise-induced changes in gene expression and their long-term impact on cancer prognosis (44, 61, 62).

Limitations and perspectives: Sample Size and Type Limitations: This study is primarily based on animal models and specific cancer cell lines, which may restrict the generalizability of the findings and their direct applicability to human cancer patients (63). While murine models provide valuable insights into tumor biology, they cannot fully replicate the complexity and heterogeneity of human tumors (64–66). Singular Focus of Study Design: Although we observed the impact of exercise on tumor growth and CD300E expression, there is a lack of exploration into variables such as exercise intensity, frequency, and duration. Moreover, the study focuses predominantly on the role of a single gene, CD300E, while tumor development involves multiple genes and signaling pathways interacting (67). Complexity in Data Interpretation: While gene expression and pathway enrichment analyses have unveiled potential biological mechanisms, the exact causal relationships remain unclear. For instance, the direct link between changes in CD300E expression and specific tumor behaviors has not been fully established. Future experimental designs should consider the effects of various types and intensities of exercise on tumor growth and how these variables interact with gene expression and immune responses within the tumor microenvironment (68). Additionally, investigating the role of CD300E across different cancers and immune backgrounds may reveal its multifunctional potential as a therapeutic target. Further mechanistic studies should delve into how CD300E activates or inhibits cancer-related pathways, particularly how it influences key tumor behaviors such as cell cycle progression, apoptosis, migration, and invasion. While current research focuses on exploring tumor therapy at the level of a single gene, future studies could use single-cell sequencing and spatial transcriptome analysis to identify a broader range of genes affected by exercise (69–72). These studies could use integrated bioinformatics approaches to elucidate gene-gene interactions and pathways co-regulated by exercise.

## 5 Conclusions

In summary, CD300E not only plays a potentially crucial role in the process of exercise-mediated tumor growth inhibition but also exhibits viability as a therapeutic target based on its expression and function across various cancers. Future research should further explore the specific molecular mechanisms of CD300E and its role in different cancers to advance the development of novel anti-cancer strategies.

## Data availability statement

The original contributions presented in the study are publicly available. This data can be found here GEO repository, accession number, GSE272213: https://www.ncbi.nlm.nih.gov/geo/query/acc.cgi?acc=GSE272213.

## Ethics statement

Ethical approval was not required for the studies on humans in accordance with the local legislation and institutional requirements because only commercially available established cell lines were used. The animal study was approved by Ethics Committees at Nanjing Medical University. The study was conducted in accordance with the local legislation and institutional requirements.

## Author contributions

ZL: Conceptualization, Data curation, Formal analysis, Funding acquisition, Investigation, Writing – original draft, Writing – review & editing. JZ: Investigation, Methodology, Project administration, Resources, Writing – original draft. RX: Investigation, Methodology, Project administration, Writing – original draft. RW: Investigation, Writing – original draft. YH: Investigation, Writing – original draft. YC: Investigation, Software, Writing – original draft, Writing – review & editing. QW: Conceptualization, Project administration, Resources, Software, Supervision, Validation, Visualization, Writing – original draft. SC (8th Author): Conceptualization, Funding acquisition, Resources, Software, Supervision, Validation, Visualization, Writing – original draft, Writing – review & editing. SC (9th Author): Funding acquisition, Project administration, Supervision, Writing – original draft, Writing – review & editing.
