# Supplementary material for: Exercise-downregulated CD300E acted as a negative prognostic implication and tumor-promoted role in pan-cancer
Source: Front Immunol. 2024 Jul 31;15:1437068. doi: 10.3389/fimmu.2024.1437068 (PMC11321962; doi:10.3389/fimmu.2024.1437068)
Supplement: Supplementary file 1 [file DataSheet_1.docx]

Supplementary Material

# Supplementary Figures and Tables

## Supplementary Figures


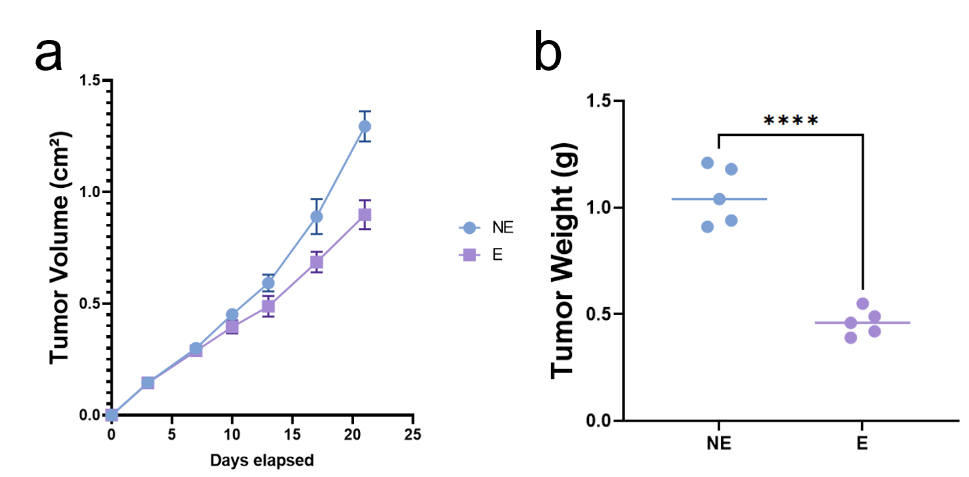


**Supplementary Figure 1.** a. Effect of CD300E on tumor volume in balb/c mice bearing 4T1 cells. b. Effect of CD300E on tumor weight in balb/c mice bearing 4T1 cells and quantitative analysis. Data presented as mean ± SD. n = 5. Significance was calculated with Student’s t test. ****P < 0.0001.


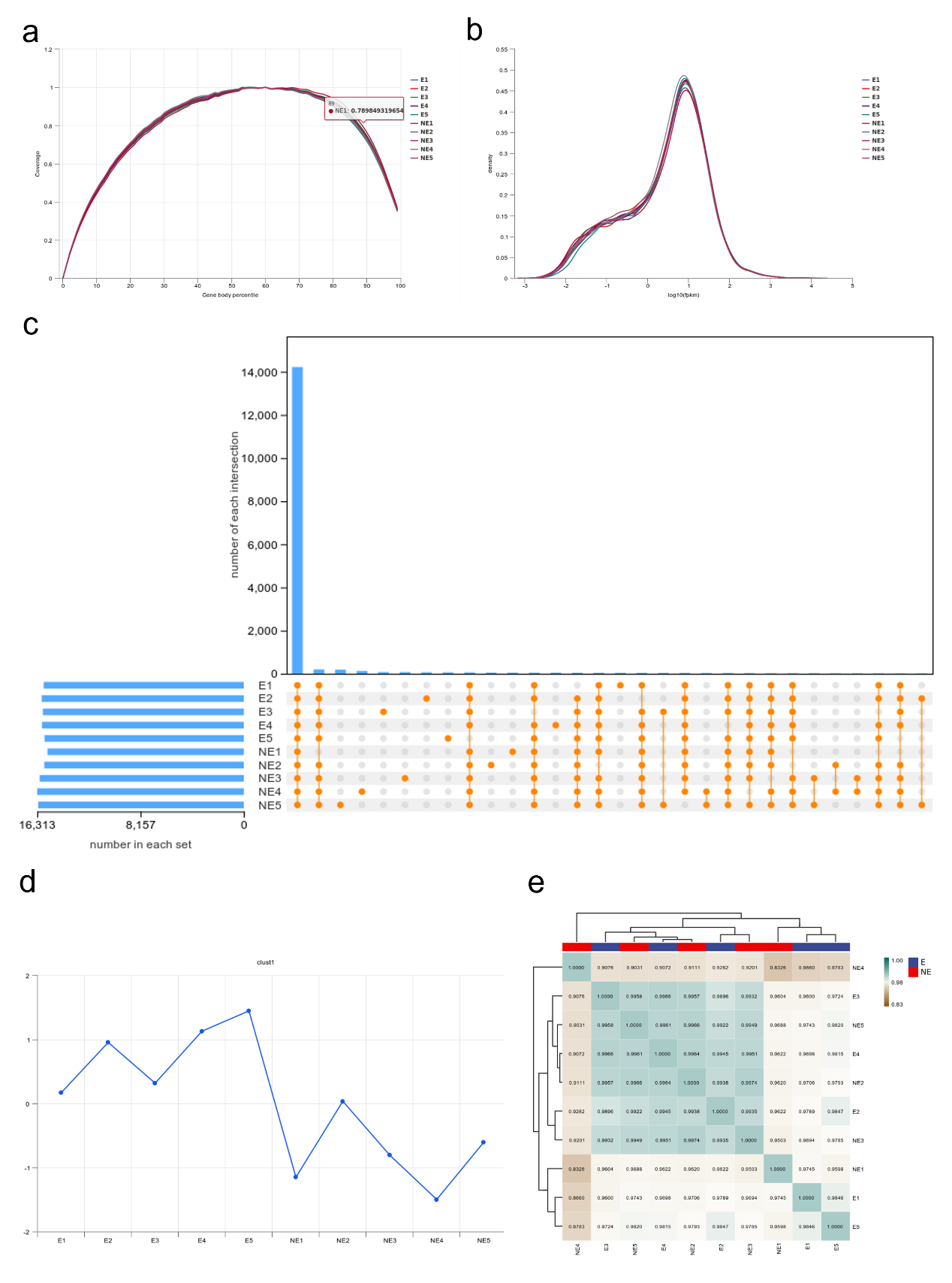


Supplementary Figure 2. a-c. Quality control of sequencing analysis. d. Comparison of gene expression levels between different groups. e. Correlation analysis between different groups.


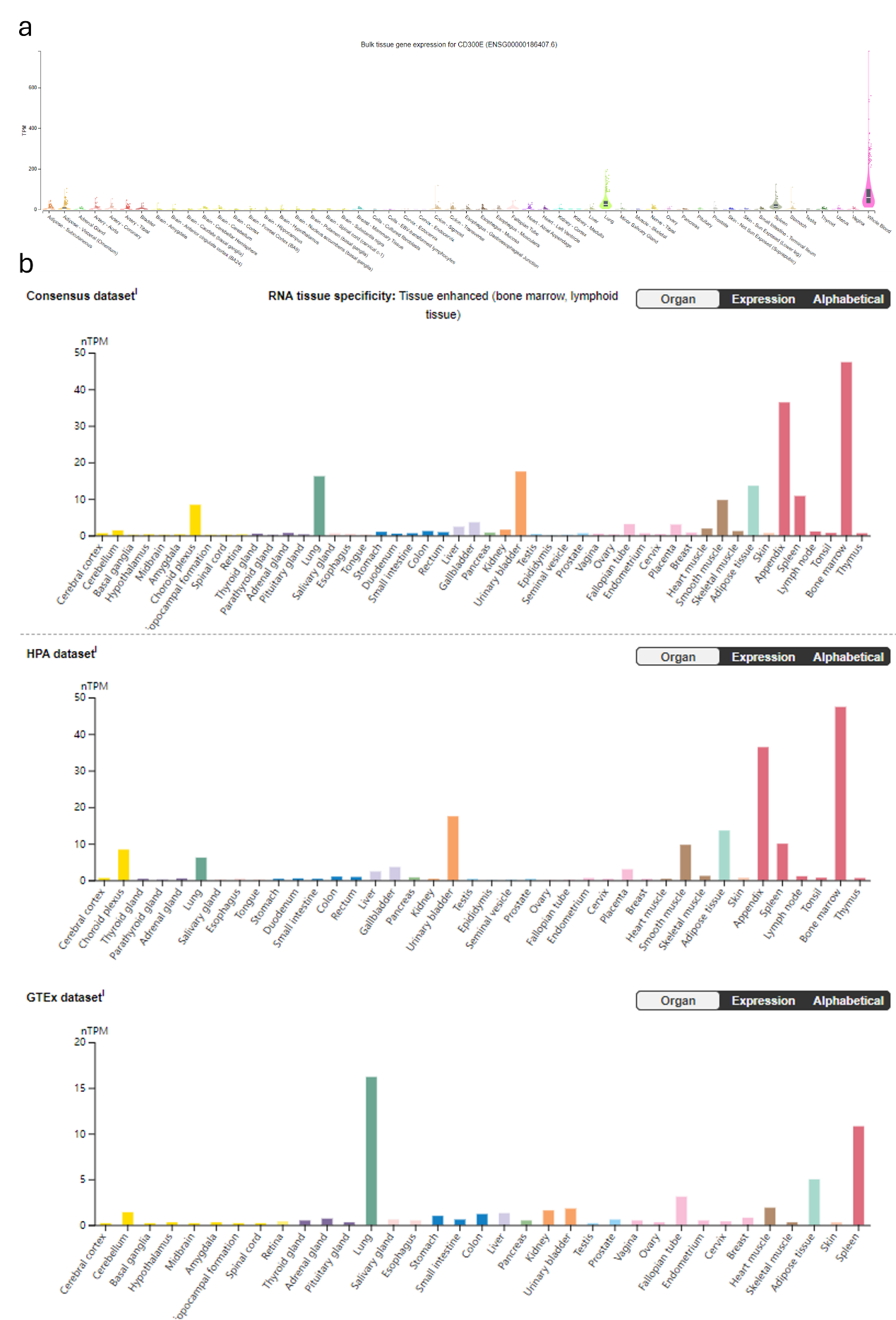


**Supplementary Figure 3.** a-b. CD300E mRNA expression in normal tissues from different datasets.


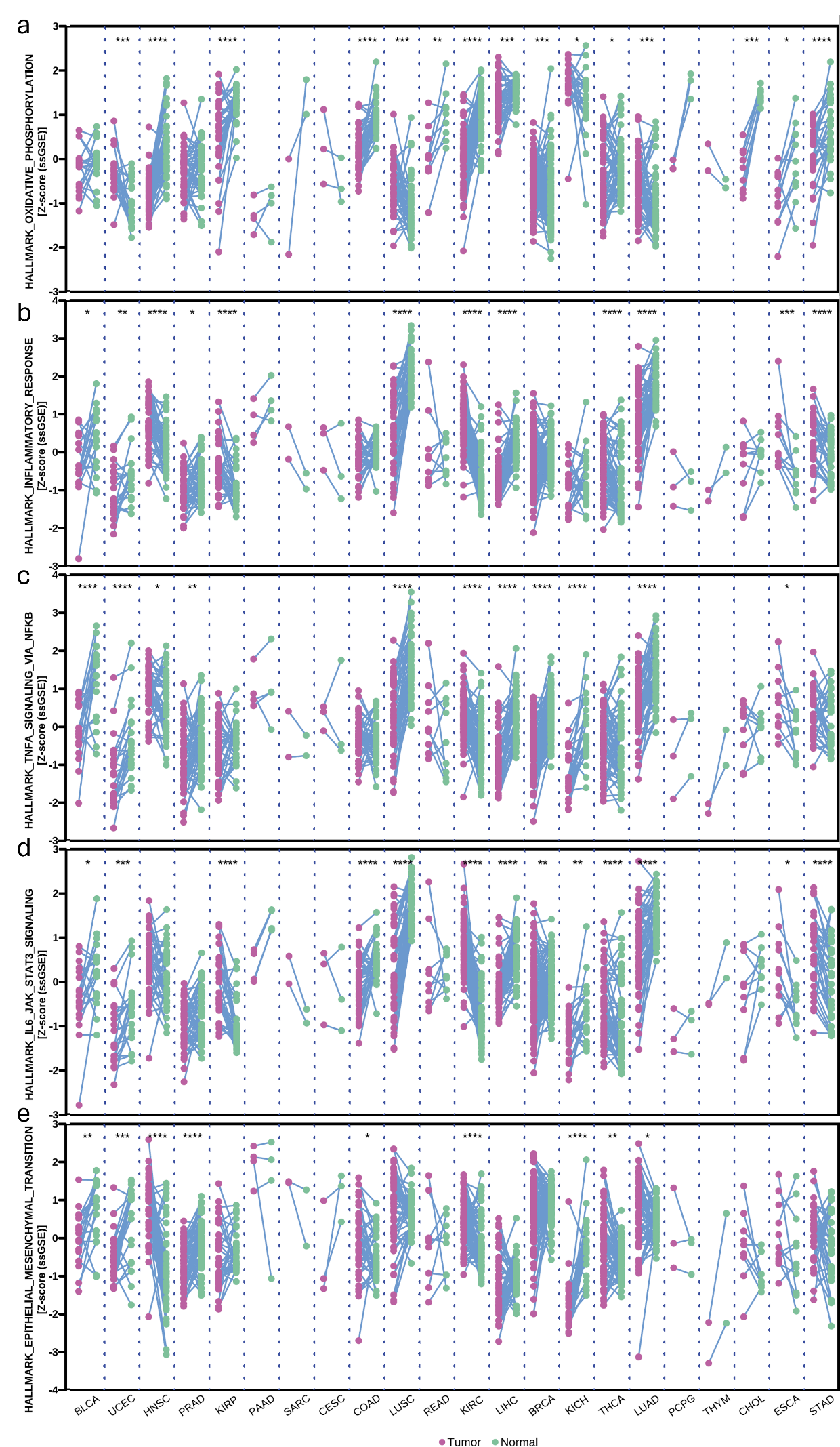


**Supplementary Figure 4**. (a-e) Violin plots demonstrate GSEA results for specific pathways, comparing gene set enrichment in tumor versus normal tissue samples. y-axis indicates the normalized enrichment score (NES) and x-axis categorizes cancer type. Color fills indicate the density of data points, with pink indicating tumors and cyan indicating normal tissue distribution.


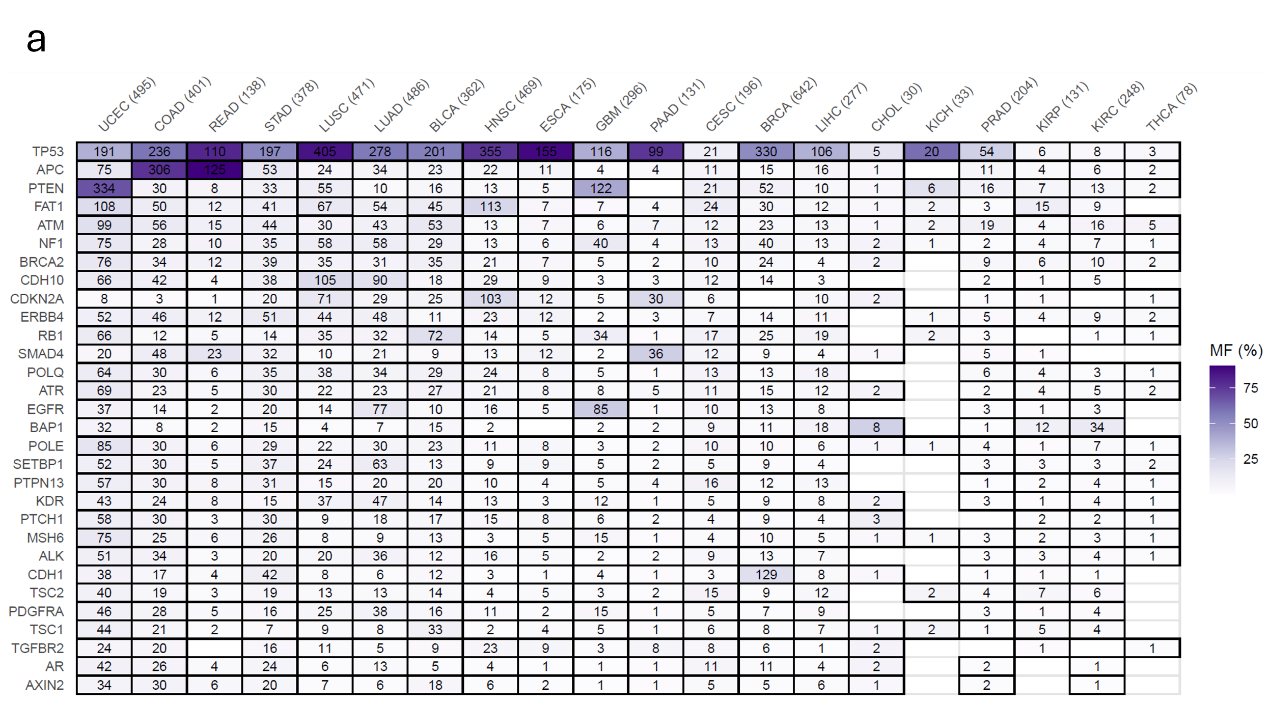


**Supplementary Figure 5**. **Mutational analysis in pan-cancer** (a). Analysis of mutation frequency and CNV in TCGA-COAD/READ. The mutation frequency of RNA modification “writers” among 20 cancer types in the TCGA cohort. The horizontal axis represents cancer types, and the number of samples is given in the parentheses. The vertical axis lists the names of the genes.


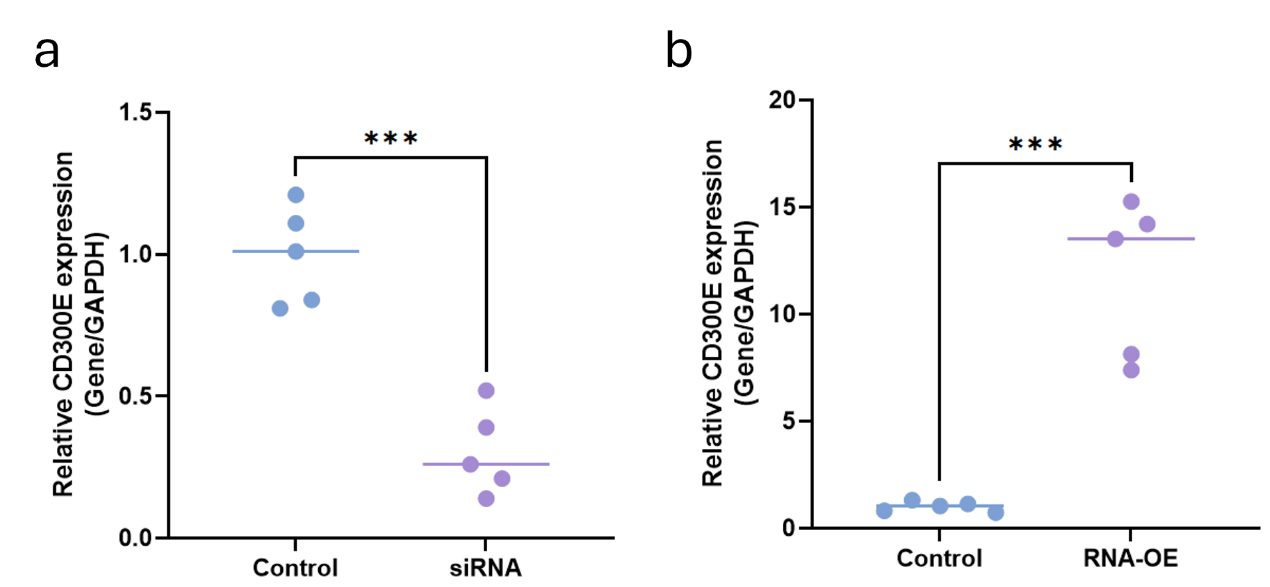


**Supplementary Figure 6**. (a-b) PCR validation of targeted gene. Significance was calculated with student t test. n = 5. ***P<0.001.

# Sup Figure 3. Schematic depiction of this work. Exercise-induce miR-133b in the circulating serum exosomes may prevent POP by down-regulating AXUD1, through which the proliferation of muscle cells was enhanced, and apoptosis was reduced.

**Supplemental Table 1. Primers for PCR used.**

| Primer Name | Primers |  |
| --- | --- | --- |
| CD300E | F: GTTTCCCCAGCAATTACAACCC  R: CAGAAGACAGCACCCAGCAT |  |
| GAPDH | F: GAAGGTGAAGGTCGGAGTCA  R: GACAAGCTTCCCGTTCTCAG |  |
| **Hairpin inserts for psiRNA-h7SK G1 (cloning sites: BbsI/BbsI) expression vector** Construct Human_cd300e #4       siRNA GC%: 38.10      Position: 236  Oligo 1  5' ACCTCGCATGTACAAGGGATATAACATCAAGAGTGTTATATCCCTTGTACATGCTT 3'  Oligo 2  5' CAAAAAGCATGTACAAGGGATATAACACTCTTGATGTTATATCCCTTGTACATGCG 3'  Construct Human_cd300e #4 \| GC%: 38.10 \| Position: 236  siRNA target:  5' GCATGTACAAGGGATATAACA 3'  siRNA scrambled:  5' GAATTAGCAGCGCAATAGATA 3' | |  |

According to

Extracellular vesicles from human plasma dampen inflammation and promote tissue repair functions in macrophages

https://www.invivogen.com/sirnawizard/construct3.php

>NM_181449.3 Homo sapiens CD300e molecule (CD300E), mRNA

ATAATCTTCATAACAATAGTCACTTATAGAAGACCTATCAGCAAATGGACTGTACTGTTCACATACAGAT
